# The role of triacylglycerols and repurposing DGAT1 inhibitors for the treatment of *Mycobacterium tuberculosis*

**DOI:** 10.1016/j.tcsw.2022.100083

**Published:** 2022-10-15

**Authors:** Alice R. Moorey, Gurdyal S. Besra

**Affiliations:** Institute of Microbiology and Infection, School of Biosciences, University of Birmingham, Birmingham B15 2TT, UK

**Keywords:** *Mycobacterium tuberculosis*, Triacylglycerols, *Tgs*

## Abstract

Latent tuberculosis poses a significant threat to global health through the incubation of undiagnosed infections within the community, and through its tolerance to antibiotics. This Special Features article explores the mechanisms by which the dormant *Mycobacterium tuberculosis* pathogen can store energy in the form of lipid inclusion bodies and triacylglycerols, which may be key in the development of novel therapeutics to treat TB.

Tuberculosis (TB) is an infection of global significance, caused by *Mycobacterium tuberculosis*, and is second only to Covid-19 in the number of deaths caused annually by a single infectious agent*.* Most cases begin with the inhalation of the bacterium, which leads to a primary infection of the lungs, although TB can also cause systemic disease. *M. tuberculosis* is remarkable in its ability to survive long periods of dormancy, during which the infection is referred to as latent TB. Several physiological and metabolic changes occur between the host and the pathogen in latent TB which lead to the formation of granulomas. The granuloma, is a hallmark of TB, typically preventing bacterial proliferation, however, it does not allow the host to completely eradicate the infection.

Briefly, when the bacterium is inhaled, it is phagocytosed by alveolar macrophages. Further immune cells are recruited to the site, while phagosomal maturation is interrupted by the bacillus, which prevents acidification of the phagosomal compartment. Macrophages constitute a large part of the granuloma, undergoing changes at the site of infection, some becoming lipid loaded foamy macrophages, others fusing to form multi-nucleated giant cells (Ramakrishnan, 2012). Further to these changes, mature macrophages in the TB granuloma can develop into epithelioid cells with an inter-linked arrangement, providing structure (Ramakrishnan, 2012). Additional immune cells are also recruited to the periphery of the granuloma, forming a diversely populated capsule with a central cavity of *M. tuberculosis* bacilli and necrotic infected macrophages, surrounded by both epithelioid and foamy macrophages, neutrophils, giant and dendritic cells, and an outer border of T- and B-cells (Fig. 1) (Ramakrishnan, 2012).

**Fig. 1:**
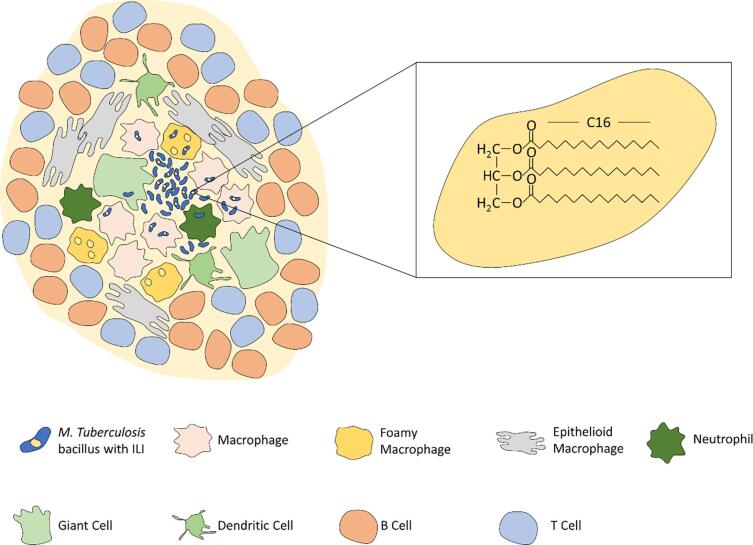
Anatomy of the granuloma. The key immune cells and pathogen cells at the site of Infection are shown in a representation of the TB granuloma. Highlighted in the box is an intracellular lipid inclusion body containing triacylglycerol, the structure of which is shown with a C16 acyl-chain length which is the preference of *M. tuberculosis.*

Granuloma formation, which restricts the growth and division of the pathogen, contributes to the clinical complication of antibiotic tolerance and resistance. Under the hypoxic conditions of the granuloma, the metabolic and phenotypic changes that occur in *M. tuberculosis* (Wayne and Hayes, 1996) render antibiotics, such as rifampicin, isoniazid, ethambutol and pyrazinamide, which target actively replicating bacteria, ineffective.

Dormant *M. tuberculosis* bacilli are faced with the challenge of fuelling the maintenance of essential cell processes in this nutrient poor environment. While encased in the granuloma, *M. tuberculosis* are able produce ATP, however the cellular levels of ATP are considerably lower than actively replicating *M. tuberculosis*. This depletion of cellular energy results in a reduction of biochemical activity within the cell which is largely responsible for the reduction in antibiotic efficiency, as the biosynthetic pathways which are targeted by antibiotics are downregulated.

Both prokaryotic and eukaryotic organisms use fatty acids as an important energy source, as they yield a higher amount of energy than other molecules, such as carbohydrates and can easily be stored within the cell (Serafim et al., 2018). *M. tuberculosis* is no different; in fact, during dormancy, the role of fatty acids for energy storage is particularly significant. Fatty acids are known to stimulate oxygen uptake in *M. tuberculosis* and can be utilised for the release of energy and carbohydrate synthesis.

Lipids such as fatty acids can be broken down through the tricarboxylic acid cycle (TCA), in which the oxidation of acetyl-CoA results in the formation of adenosine triphosphate (ATP) and carbon dioxide. Additionally, the glyoxylate shunt is a pathway through which acetyl-CoA is converted to a dicarboxylic acid, succinate, which is involved in the synthesis of carbohydrates. These two cycles are sufficient when run in parallel for fatty acids to be used as the main carbon source, which helps cells endure hypoxic environments.

Moreover, for efficient storage, fatty acids are accumulated as triacylglycerols (TAGs) in the adipose tissue of most mammals and in aggregated lipids within bacterial cells, known as intracellular lipid inclusion (ILI) bodies (Serafim et al., 2018). The amount of lipid inclusion bodies in the cytoplasm of *M. tuberculosis* increases during cell dormancy, and accumulation of TAG can be induced under hypoxic environments and nitrogen deprivation, suggesting a key role in *M. tuberculosis* survival within the host (Sirakova et al., 2006, Santucci et al., 2019). A protein identified in *Mycobacterium bovis* BCG, BCG_1721, has bi-functional activity for both the accumulation and hydrolysis of TAG and is important for the growth of cells following resuscitation (Low et al., 2010). In addition to intracellular TAG, there is strong evidence that both diacylyglycerol (DAG) and TAG are present in the outer membrane of mycobacterial species. The export of TAG from the cytoplasm to the outer membrane by an efflux pump (Rv1410) and lipoprotein LprG has been observed, which supports the theory that TAG provides an undefined function to the complex *M. tuberculosis* cell wall (Martinot et al., 2016, Viljoen et al., 2016).

The synthesis of diacylglycerol (DAG) is the first committed step in mycobacterial TAG synthesis. The PlsB/PlsC acyltransferases sequentially esterify glycerol-3-phosphate resulting in phosphatidic acid, which is dephosphorylated by a phosphatidic acid phosphatase and used in the synthesis of DAG and TAG. Fifteen genes have been identified in *M. tuberculosis* as encoding putative triacylglycerol synthase (Tgs) enzymes which are responsible for acylating DAG using acyl-CoAs (Fig. 2, Table 1) (Daniel et al., 2004). When expressed in *Escherichia coli*, the *tgs* activity of the cell lysate increased for all expressed proteins compared to wild type *E. coli*, and the four Tgs proteins with highest *tgs* activity were designated Tgs1 (Rv3130c), Tgs2 (Rv3734c), Tgs3 (Rv3234c) and Tgs4 (Rv3088) (Daniel et al., 2004).

**Fig. 2:**
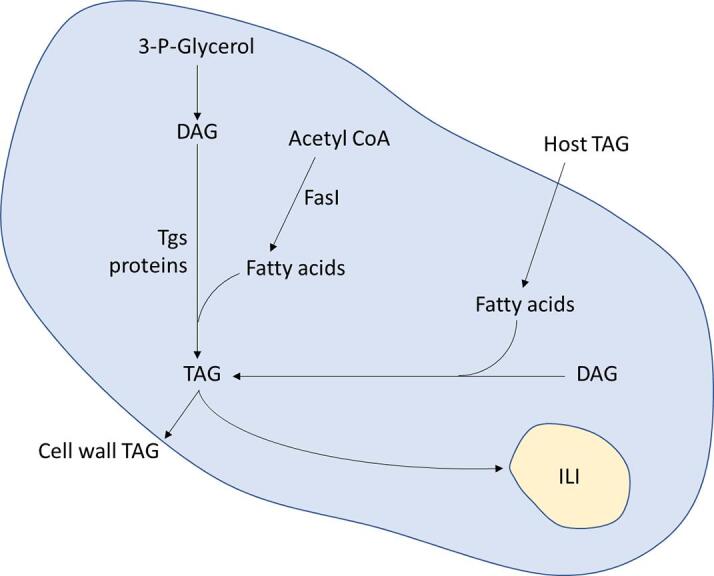
Triacylglycerol synthesis in *M. tuberculosis.* There are two pathways by which Mycobacteria are able to accumulate TAG: either through *de novo* synthesis, or by utilizing host TAG which is imported into the cell as fatty acids.

**Table 1:** Confirmed and putative *tgs* genes in *M. tuberculosis* H37Rv*.* Sub-cellular location is recorded where known. All 15 *tgs* genes are considered non-essential for *in vitro* growth in rich media (Dejesus et al., 2017) however essentiality has been observed in specific conditions for certain genes. ^1^Rengarajan et al., 2005; ^2^Tsolaki et al., 2004; ^3^Sassetti et al., 2003.

Gene name	Notes on essentiality
Rv3130c (*tgs1*)	
Rv3734c (*tgs2*)	
Rv3234c (*tgs3*)	
Rv3088 (*tgs4*)	
Rv0221	
Rv0895	Essential in primary murine macrophages^1^
Rv1425	
Rv1760	Non-essential, deletion observed in clinical isolates^2^
Rv2285	
Rv2484c	
Rv3087	Essential in C57BL/6J mouse spleen^3^
Rv3233c	
Rv3371	
Rv3480c	Essential in primary murine macrophages^1^
Rv3740	

*M. tuberculosis* has a group of 48 genes known as the DosR dormancy regulon, a regulatory system which is activated under conditions of hypoxia. Tgs1 is a powerfully inducible gene located within the DosR region and is upregulated in *M. tuberculosis* clinical isolates (Chauhan and Tyagi, 2009). The involvement of Tgs1 in virulence and the rapid formation of necrotising granulomas has been demonstrated in zebra fish, and the deletion of Tgs1 is known to cause a dramatic (but not complete) reduction in TAGs (Sirakova et al., 2006, Daniel et al., 2011). Tgs1 has directly been linked to the accumulation of TAG in ILIs and shows preference for long-chain fatty acids for TAG synthesis (Sirakova et al., 2006, Viljoen et al., 2016). Both the synthesis of TAG and the accumulation of TAG are relatively conserved among species of *Actinobacteria*. The formation of ILIs occurs in many species of the *Mycobacterium* genus, including but not limited to *Mycobacterium leprae*, *Mycobacterium avium* and *Mycobacterium abscessus* (Santucci et al., 2019)*.*

During latent TB infections, *M. tuberculosis* is not alone in accumulating TAG; when incubated under hypoxic conditions, host macrophages, including human peripheral blood monocyte-derived macrophages and THP-1 derived macrophages, accumulate lipid droplets (Daniel et al., 2011). A remarkable feature of the *M. tuberculosis* dormancy phenotype is its ability to exploit the host’s response to hypoxia by utilising host TAG. Dual isotope labelling of macrophage TAG using triolein [glycerol-1,2,3-3H, carboxyl-1-14C] has revealed that the bacterium is able to import host derived fatty acids from TAG, which are then incorporated into mycobacterial TAG molecules and are accumulated within intracellular lipid droplets (Daniel et al., 2011).

There are two enzymes involved in the synthesis of human TAG: diacylglycerol O-acyltransferase 1 (DGAT1) and DGAT2 which catalyse the synthesis of TAG from DAG and long-chain fatty acyl-CoAs (Oelkers et al., 1998). Both enzymes are membrane bound acyltransferases and have been implicated in metabolic diseases, cancer progression and obesity. Up-regulation of DGAT1 in cancer tissues has been associated with poor patient outcomes, while DGAT1 knock-out mice are less susceptible to diet induced obesity (Smith et al., 2000, He et al., 2014). DGAT1 is essential for lipid accumulation in TB infected macrophages, which suggests that inhibiting DGAT1 could lead to a reduction in TAG availability in TB infections. These mechanisms allow *M. tuberculosis* to survive using TAG as an energy source and could yield potential novel targets for TB therapeutics and have led us to consider the possibility of exploiting DGAT1 inhibitors for the treatment of latent TB.

The proven viability of DGAT1 null mice initially led to an increase in research into DGAT1 inhibitors for obesity related diseases, but there have since been DGAT1 inhibitors developed for a variety of medical uses, from type 2 diabetes to tumour suppression (Smith et al., 2000). Niacin, also known as nicotinic acid or Vitamin B3, has long been associated with *M. tuberculosis,* since it was discovered that the bacterium secretes the compound, and a test was developed to distinguish mycobacteria based on niacin detection. Interestingly, Niacin has been shown to reduce TAG synthesis in human cells through the competitive inhibition of DGAT2, but not DGAT1 (Ganji et al., 2004).

## Conclusion

Latent TB poses a significant threat to global health through the incubation of undiagnosed infections within the community, and through its tolerance to antibiotics. Tackling the mechanisms by which the dormant pathogen can store energy may be key in the development of novel therapeutics to treat TB. A two-pronged approach of inhibiting *de novo* TAG synthesis in *M. tuberculosis* and preventing the uptake of host TAG is essential and could dramatically reduce the ability of *M. tuberculosis* to persist within the granuloma. Fortunately, the secretion of niacin by the bacterium prevents one route of host TAG synthesis, *via* the inhibition of DGAT2. Looking to the future of TB drug discovery, known inhibitors of DGAT1 should be screened against *M. tuberculosis* to establish whether any existing drugs could be exploited as anti-mycobacterial agents.

## Ethics Statement

No ethical issues to report.

## CRediT authorship contribution statement

**Alice R. Moorey:** Conceptualization, Writing – original draft, Writing – review & editing. **Gurdyal S. Besra:** Conceptualization, Writing – original draft, Writing – review & editing.

## Declaration of Competing Interest

The authors declare the following financial interests/personal relationships which may be considered as potential competing interests: Gurdyal Singh Besra reports financial support was provided by Medical Research Council. Gurdyal Singh Besra reports a relationship with Microbiology Society that includes: travel reimbursement.
